# A Flare of Systemic Lupus Erythematosus Disease After COVID-19 Infection: A Case of Lupus Cerebritis

**DOI:** 10.7759/cureus.16104

**Published:** 2021-07-02

**Authors:** Muhammad Zain Khalid, Sylvette Rogers, Ayesha Fatima, Manal Dawe, Romil Singh

**Affiliations:** 1 Internal Medicine, Liaquat National Hospital and Medical College, Karachi, PAK; 2 Family Medicine, Caribbean Medical University, Des Plaines, USA; 3 Gynaecology & Obstetrics, Fauji Foundation Hospital, Rawalpindi, PAK; 4 Internal Medicine, Capital Medical University, Beijing, CHN; 5 Critical Care, Mayo Clinic, Rochester, USA

**Keywords:** systemic lupus erythematosus disease, covid-19, sars-cov-2, sle, lupus cerebritis

## Abstract

The association between coronavirus disease 19 (COVID-19) and autoimmune disease has been mounting, and literature on COVID-19-induced flare-up of systemic lupus erythematosus (SLE) disease is lacking. We describe a case of lupus cerebritis triggered by COVID-19 in a young female with SLE, who presented with fluctuated mentation, psychomotor retardation, slow speech, and intermittent choreiform movement in the upper part of the body. She had a history of COVID-19 infection three weeks back. Her serum immunoglobulin G antibodies were positive against COVID-19. On examination, she had psychomotor agitation, intermittent choreiform movements of upper limbs, and poor speech. Brain magnetic resonance imaging revealed hyperintense signals in the white matter of both hemispheres, suggestive of lupus cerebritis secondary to COVID-19 infection and lack of any other identifiable risk factor. Management included methylprednisolone, prednisone, and olanzapine. The patient was also placed on monthly intravenous cyclophosphamide, and her condition started improving gradually.

## Introduction

Severe acute respiratory syndrome coronavirus 2 (SARS-CoV-2) is the causative agent of coronavirus disease 19 (COVID-19). COVID-19 has a broad spectrum of clinical manifestations involving different systems of the body [1]. COVID-19 generally presents with signs and symptoms of the respiratory system, including flu-like illness complicated by acute respiratory distress syndrome (ARDS) and lung failure [2]. Other manifestations and complications include severe metabolic syndrome, acute kidney injury, neurological syndromes, cardiovascular and thromboembolic events such as encephalopathy, seizures, and stroke [3-7]. A possible association between COVID-19 and autoimmune disease has also been reported in many case reports [8]. Systemic lupus erythematosus (SLE) disease has been reported in patients with COVID-19 [9]. Herein, we describe a rare case of lupus cerebritis triggered by SARS-CoV-2 in a young female diagnosed with SLE.

## Case presentation

A 29-year-old female with a past medical history of SLE was brought to the emergency department with fluctuated mentation, fatigue, anorexia, and psychomotor retardation for the last week. She also complained of incoherent speech and intermittent choreiform movement in the upper part of the body. She was diagnosed with SLE four years back, having urticaria and erythematosus rash with itching, scaling of the palm of hands, and hyperkeratosis of the sole, for which she was taking hydroxychloroquine and prednisone. She was admitted to the hospital three weeks back due to worsening dyspnea, fever, and cough. She had tachypnea, wheezing, and a chest X-ray revealed diffuse infiltrates in both lungs. Her COVID-19 polymerase chain reaction (PCR) test was positive, and she was commenced on azithromycin and 6mg dexamethasone for five days. Her condition improved gradually, and she was discharged six days later.

On clinical examination, she looked anxious with poor speech. She had a temperature of 99^o^F, respiratory rate of 23/minute, heart rate of 87/minute, blood pressure of 110/70 mmHg, and oxygen saturation of 96%. Her cardiovascular and respiratory examination was unremarkable, with normal vesicular breathing and heart sounds. Neurological examination revealed psychomotor agitation, intermittent choreiform movements of upper limbs, and poor speech. She had no signs of meningeal irritation, muscle strength loss, seizure episodes, and any history of trauma and illicit drug use. Her repeat COVID-19 PCR test was negative; however, serum immunoglobulin G (IgG) antibodies were positive against COVID-19.

**Figure 1:**
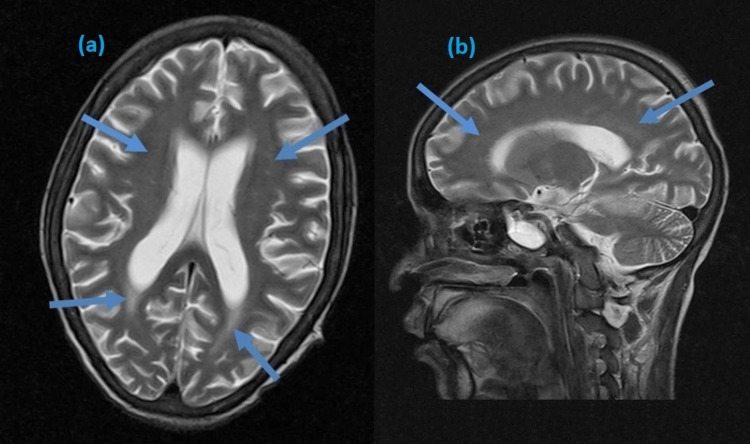
Brain MRI showing hyperintense signals in the temporal and parietal regions in axial (a) and sagittal (b) planes

Her initial blood investigations revealed thrombocytopenia and mild elevation of creatinine (Table 1). Infectious workup was negative for any organism. The urine screen was negative for any illicit drug use, and the result of her repeat autoimmune screening is shown in Table 2. Her brain magnetic resonance imaging (MRI) revealed hyperintense signals in the bilateral parietal and temporal lobes, suggestive of lupus cerebritis (Figure 1). She was diagnosed with lupus cerebritis, an exacerbation of SLE due to COVID-19 infection. CSF analysis was not performed because she refused lumbar puncture.

**Table 1:** Initial blood investigations

Parameter	Lab value	Reference range
White blood cell count	10,600	4,000-11,000 mm^3^
Platelet count	135,000	150,000-350,000 mm^3^
Red blood cell count	3.9	04-06 million cells/mm^3^
Hemoglobin	11.9	11.5-17.5 mg/dL
Erythrocyte sedimentation rate	23	0-20 mm/hour
C-reactive protein	11	< 10 mg/L
Creatinine	1.4	0.9-1.1 mg/dL
Blood urea nitrogen	23	18-45 mg/dL
Aspartate aminotransferase	33	08-35 IU/L
Alanine aminotransferase	29	10-40 IU/L
Glycosylated hemoglobin	6-1	5.7%-6.4%

**Table 2:** Autoimmune screening for SLE Anti-dsDNA: anti-double-stranded deoxyribonucleic acid, Ab: antibody, Anti-CCP: anti-cyclic citrullinated peptides, Anti-β2GP1: anti-beta-2 glycoprotein 1

Parameter	Lab value	Reference range
Complement C3 protein	88	90-10 mg/dL
Complement C4 protein	10	10-40 mg/dL
Anti-dsDNA Ab	55	< 35 IU/mL
Anti-CCP Ab	Negative	Negative
Anticardiolipin Ab	Negative	Negative
Lupus anticoagulant	Negative	Negative
anti-β2GP1 Ab	Negative	Negative

Her initial management included 1g methylprednisolone for three consecutive days, 30mg of prednisone daily, and 5mg of olanzapine daily for two weeks. The patient was also placed on monthly 1g intravenous cyclophosphamide. Her condition started improving gradually, and she was stable on day six of hospitalization. She was discharged on maintenance oral steroid therapy with follow-up.

## Discussion

SLE is a multisystem disease and has complex clinical manifestations involving several organs in the body such as the heart (lupus carditis), kidney (lupus nephritis), brain (lupus cerebritis), skin, eyes, joints, and muscles [10]. SLE is an autoimmune disease characterized by the production of pathognomonic autoantibodies. Many viruses have been implicated in the etiology of SLE [11]. Epstein-Barr virus, cytomegalovirus, retroviruses, and parvovirus B19 are the possible triggers of SLE [10,11]. SLE triggered by COVID-19 has also been reported in many cases [12]. SLE patients are at increased risk of infections due to prolonged use of immunosuppressive drugs and autoimmune syndromes. Infections can exacerbate SLE activity and are reported to be a significant cause of death among SLE patients (37.7%) [13]. SLE patients are susceptible to COVID-19 infection and can worsen the activity of SLE. Alharthy et al. reported a case of COVID-19 infection with a flare of SLE [14]. Our patient also had SLE and presented with neuropsychiatric manifestations (lupus cerebritis) triggered by COVID-19.

COVID-19 is an inflammatory disease leading to a widespread immune response throughout the body. Literature is lacking on the association between COVID-19 and SLE and the flare of SLE. Several studies have linked COVID-19 with other autoimmune diseases such as rheumatoid arthritis and multiple sclerosis. Joo et al. highlighted a link between exposure to coronavirus, parainfluenza, and metapneumovirus for six to seven weeks and increased the incidence of rheumatoid arthritis [8]. Lupus cerebritis due to COVID-19 may be due to angiotensin-converting enzyme 2 (ACE 2) receptors in the central nervous system, including basal ganglia, hippocampus, and glial cells. SARS-CoV-2 induces a severe immune response, resulting in the release of inflammatory and pro-inflammatory cytokines and chemokines, disrupting the blood-brain barrier and leading to neuropsychiatric manifestations [2]. Another mechanism may be microangiopathy arising from cytokine storm and complement activation leading to cerebral ischemia and microinfarction [12]. The cytokine storm induced by SARS-CoV-2 leads to immune-mediated damage to neuronal cells by molecular mimicry such as the production of autoantibodies like antinuclear antibodies (ANAs) [14].

The diagnosis of lupus cerebritis is based on clinical presentation, serum chemistry (complete blood count), immunological studies (ANAs and anti-dsDNA antibodies), and brain imaging using computed tomography and MRI [15]. Cerebrospinal fluid analysis is occasionally done to rule any infectious cause of neuropsychiatric manifestations. Electroencephalography has a low sensitivity and specificity for diagnosis. Management depends on the severity of the disease [15]. For mild to moderate illness, glucocorticoids in combination with azathioprine or mycophenolate are used. Intravenous cyclophosphamide can be added in severe cases [16]. Antimalarials are used as alternatives to steroids or as supplements to accelerate the steroid taper. In resistant cases, rituximab, plasmapheresis, and intravenous immunoglobulins can be used. Antipsychotics such as olanzapine can be used in case of severe psychosis [16].

## Conclusions

Our patient was a known case of SLE. Her neuropsychiatric manifestation following COVID-19 infection, findings on brain MRI, and the improvement in her condition on starting appropriate treatment justify the flare of SLE as lupus cerebritis secondary to SARS-CoV-2. Our case describes the possible role of COVID-19 in the flare of SLE, albeit single case observations have limitations. There is a dire need for data to support this hypothesis, and more cases of autoimmune disorders are required to declare themselves following COVID-19 infection.
